# Myocardial tissue characterization in patients with hereditary gelsolin (AGel) amyloidosis using novel cardiovascular magnetic resonance techniques

**DOI:** 10.1007/s10554-019-01570-4

**Published:** 2019-03-08

**Authors:** Lauri Lehmonen, Touko Kaasalainen, Sari Atula, Tuuli Mustonen, Miia Holmström

**Affiliations:** 1 HUS Medical Imaging Center, Radiology, University of Helsinki and Helsinki University Hospital, Haartmaninkatu 4, PO Box 340, 00029 HUS Helsinki, Finland; 2 Department of Physics, University of Helsinki, Helsinki, Finland; 3 Clinical Neurosciences, Neurology, University of Helsinki and Helsinki University Hospital, Helsinki, Finland; 4 Faculty of Medicine, University of Helsinki, Helsinki, Finland

**Keywords:** Cardiovascular magnetic resonance, Amyloidosis, Feature tracking, Tagging, T1 mapping, Extracellular volume

## Abstract

Gelsolin (AGel) amyloidosis is a hereditary condition with common neurological effects. Myocardial involvement, especially strain, T1, or extracellular volume (ECV), in this disease has not been investigated before. Local myocardial effects and possible amyloid accumulation were the targets of interest in this study. Fifty patients with AGel amyloidosis were enrolled in the study. All patients underwent cardiovascular magnetic resonance imaging, including cine imaging, T1 mapping, tagging, and late gadolinium enhancement (LGE) imaging at 1.5 T. Results for volumetry, myocardial feature-tracking strain, rotation, torsion, native T1, ECV, and LGE were investigated. The population mean native T1 values in different segments of the left ventricle (LV) varied between 1003 and 1080 ms. Myocardial mean T1 was 1031 ± 37 ms. T1 was highest in the basal plane of the LV (1055 ± 40 ms), similarly to ECV (30.0% ± 4.4%). ECV correlated with native T1 in all LV segments (p < 0.005). Basal LGE was detected in 76% of patients, and mid-ventricular LGE in 32%. LV longitudinal strain was impaired (− 17.4% ± 2.6%), significantly decreasing apical rotation (p = 0.018) and concurrently myocardial torsion (p = 0.005). LV longitudinal strain correlated with mean T1 and ECV of different LV planes (p < 0.04; basal p < 0.01). Myocardial involvement in AGel amyloidosis is significant, but the effects are local, focusing on the basal plane of the LV.

## Introduction

Hereditary gelsolin (AGel) amyloidosis is a Finnish dominantly inherited systemic amyloidosis. Neurological characteristics of the disease include cranial and peripheral neuropathy, corneal dystrophy, and skin disease [1, 2]. Several myocardial manifestations, such as cardiac arrhythmia and atrioventricular blocks, have also been described in this patient population [3]. Amyloidosis, in general, often results in diffuse myocardial involvement [4]. The disease involves deposit of abnormal proteins, i.e. amyloids, in multiple organs of the body, causing disruption of tissue and function [5].

Cardiovascular magnetic resonance (CMR) imaging is a valuable clinical tool in diagnosis of myocardial diseases. Unlike other imaging modalities, CMR enables characterization of tissue composition using fundamental magnetic properties. Combining this capability with the possibility for regional motion analysis makes CMR the top choice for repeatable, investigational use.

CMR facilitates non-invasive and repeatable quantitative tissue characterization through relaxation time mapping, myocardial strain analysis, and late gadolinium enhancement (LGE). The present study was designed to evaluate and characterize cardiac involvement in patients with AGel amyloidosis. We used novel and conventional CMR methods, including T1 mapping, extracellular volume (ECV) analysis, LGE imaging, tagging, and feature tracking (FT).

## Methods

### Study population

Fifty patients with Finnish hereditary AGel amyloidosis were included in this prospective study. Patients were selected from the patient registry of Finnish Gelsolin amyloidosis (FIN-GAR). Patients selected from the registry had a biopsy and genetic testing. All Finnish patients to date have been confirmed to carry gelsolin gene mutation G654A. The disease penetrance is 100%, meaning all patients show symptoms of the condition. Mean age of the patients was 66 ± 7 years. Exclusion criteria for the study population were (1) age below 50 years, (2) implanted cardiac pacemaker, (3) claustrophobia, and (4) implanted metal objects that could interfere with CMR. Patients selected were older than 50 years due to disease progression beginning at an older age [1].

### CMR protocol and data analysis

All patients underwent CMR scan using a 1.5 T Avanto^fit^ system (Siemens Healthineers, Erlangen, Germany). The scans were performed using a 32-channel cardiac receiver coil with retrospective electrocardiographic gating. Images were acquired in breath-hold to minimize breathing artifacts. The scan protocol included stacks of balanced steady-state free precession (bSSFP) cine images with 30 temporal phases in short-axis direction and a 4-chamber stack in long-axis direction. Pre-contrast and post-contrast shortened modified look-locker inversion recovery (ShMOLLI; TR/TE 2.1/1.1 ms, angle 35° and 8 mm slice thickness) T1 mapping, tagging, and inversion recovery spoiled gradient echo LGE images were acquired in the same basal, mid-ventricular, and apical planes. Gadolinium-based contrast agent (gadoterate meglumine, Dotarem) of 0.2 mmol/kg was used as a contrast agent. BSSFP-cine images, pre-contrast T1 mapping, and spatial modulation of magnetization (SPAMM) tagging were acquired prior to contrast agent administration. LGE images were acquired 5 min and post-contrast T1 mapping 12 min after the contrast agent administration. Cine images were analyzed for volumetric data and strain data. The volumetric analysis was performed using QMass MR software v7.6 (Medis Medical Imaging Systems, Leiden, The Netherlands). FT strain, tagging, T1, and ECV analysis were performed with Segment v2.2 R6190 (Medviso AB, Lund, Sweden) [6–8]. The strain module of Segment employs a non-rigid elastic registration-based algorithm with limited memory optimizer for strain quantification. LGE images were analyzed visually and computationally with QMass, using a full width at maximum method with a 50% threshold. Left-ventricular (LV) short-axis strain, T1, ECV, and LGE results were all recorded according to AHA 17-segment model [9]. Right-ventricular (RV) and longitudinal values were collected as means.

### Feature tracking and tagging analysis

**Fig. 1:**
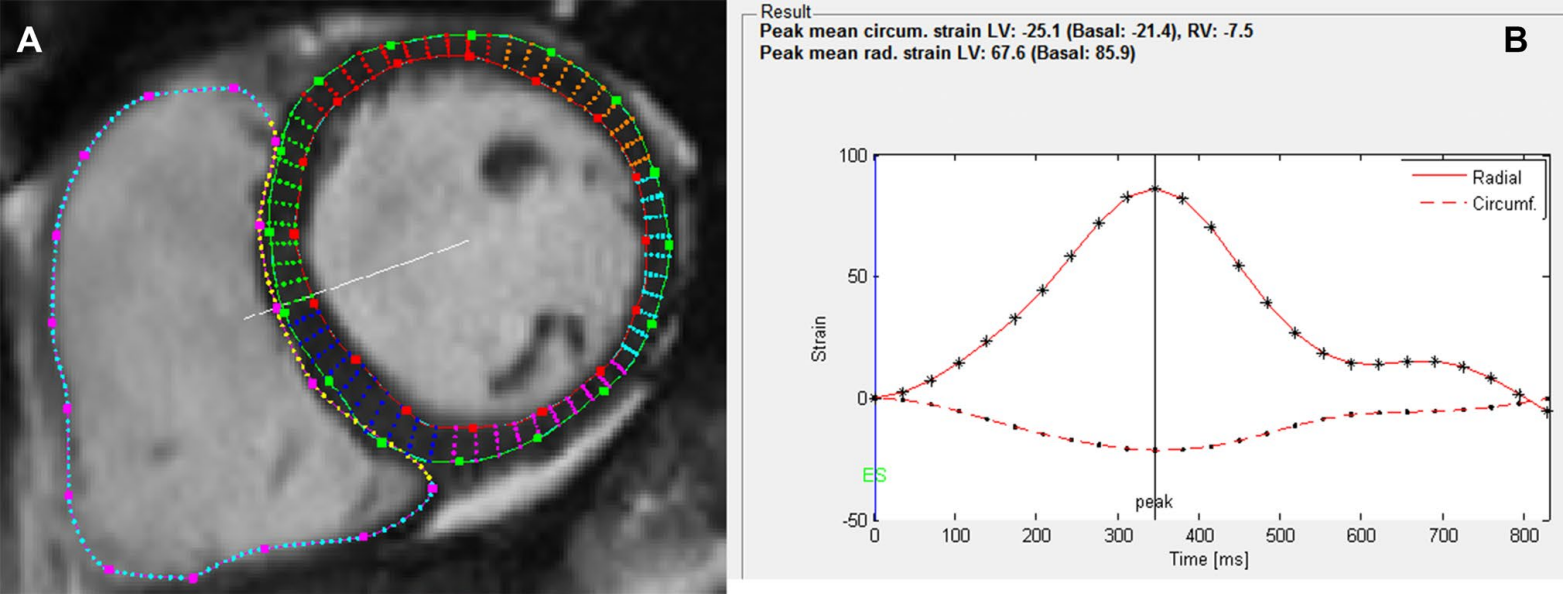
**a** Basal short-axis view of the left and right ventricles at end diastole. LV epicardial and endocardial borders as well as RV endocardial borders have been manually drawn in the image. Points along the borders allow manual correction of the segmentation. **b** The resulting strain curves for mean LV circumferential (dashed line) and radial strain (solid line). Different markers along the curves (. and *) correspond to different time points in the cine image sequence, i.e., there are 25 temporal phases

Basal, mid-ventricular, and apical short-axis and 4-chamber long-axis cine images were selected for FT analysis at end-diastole according to Taylor et al. [10]. Epicardial and endocardial borders of the LV, and endocardial border of the RV were manually drawn in the end-diastolic images using the manual segmentation tools of Segment software. The rest of the image sequence was segmented automatically and manually corrected if needed. The resulting strain curves (Fig. 1) were exported from Segment, and used for the calculation of peak strain, peak systolic strain rate, and peak diastolic strain rate with MATLAB R2017A (The MathWorks, Inc., Natick, MA, USA). Strain and strain rate values were collected in circumferential, radial, and longitudinal directions.

Tagging images were used to derive peak apical and basal rotation and peak LV myocardial torsion. Torsion is defined as the difference between apical and basal rotation, normalized with the long-axis distance between the slices and mean radius of the slices. Tagging images were acquired in addition to cine images since myocardial rotation is best recorded with the tagging grid pattern. In the normal cine images, the myocardial wall is homogeneous and rotational analysis is unreliable; rotation results have been shown to have no acceptable agreement between tagging and FT [11]. Tagging analysis was performed in the same way as the FT analysis.

### T1 and ECV analysis

**Fig. 2:**
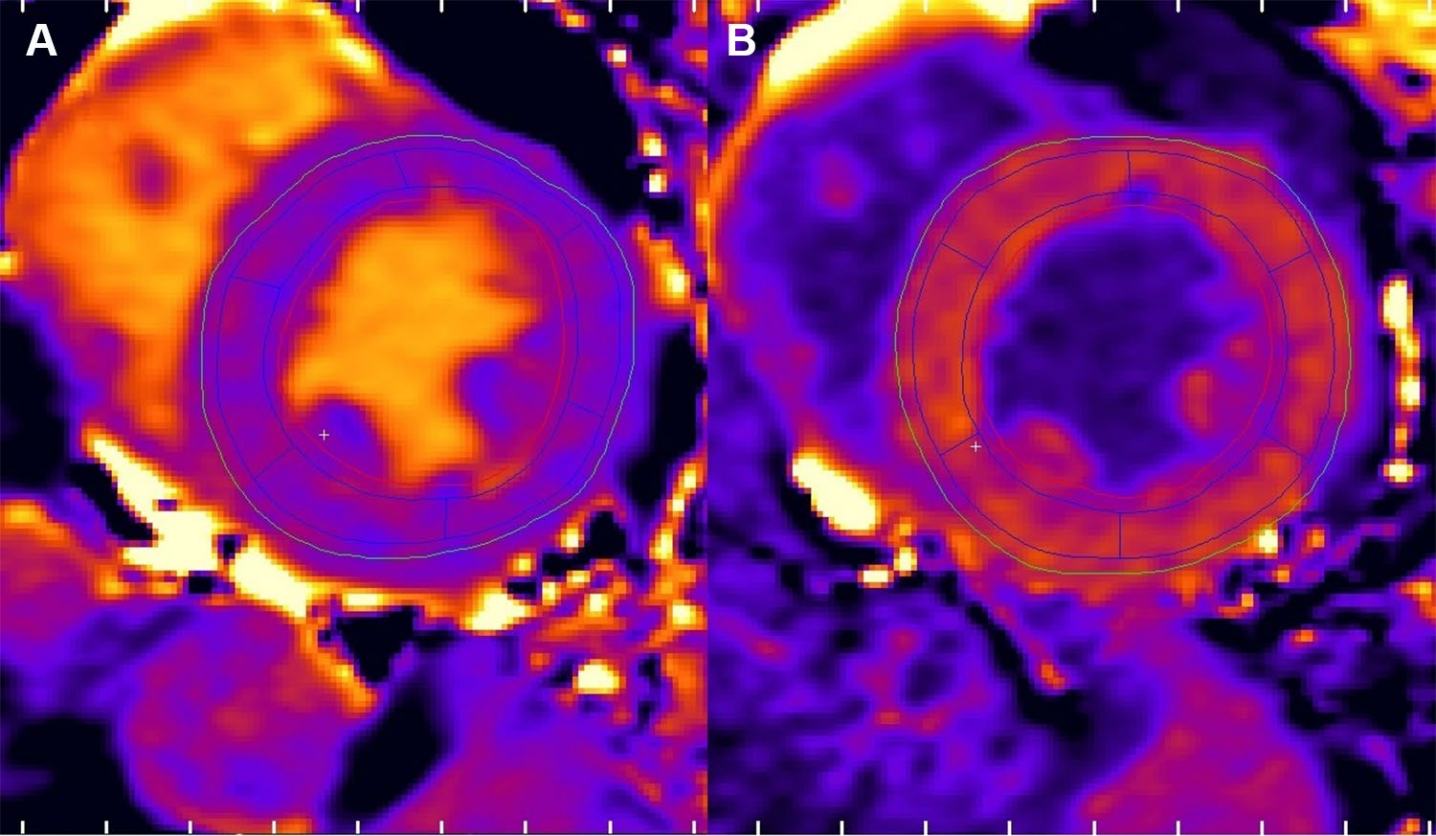
Layout of the T1 and ECV analysis. **a** mid-ventricular pre-contrast T1 map with six ROI segments. **b** corresponding post-contrast T1 map

The T1 images were used to derive pre-contrast and post-contrast T1 maps. The motion corrected pre-contrast ShMOLLI images were first loaded into Segment. Then, epicardial and endocardial borders were imported from the FT analysis to the T1 images, the segmentation was verified visually, and corrected manually if needed. The software was used to generate pre-contrast and post-contrast T1 maps. Regions of interests (ROIs) were placed on the T1 maps according to the AHA 17-segment model using the built-in tool to match the segments of the strain analysis. The ROIs were cropped 20% (default value) from the epicardial and endocardial borders to avoid artifacts. ROIs placed in the pre-contrast T1 maps were copied to the post-contrast maps (Fig. 2). ECV was calculated with the same software using the ROIs of the T1 maps and with an additional ROI in the blood pool. Hematocrit was acquired via blood test directly after the CMR study.

### Statistical analysis

Statistical analysis was performed using IBM SPSS Statistics version 24 for Windows (IBM Corp., Armonk, NY, USA). Results are reported as mean ± standard deviation. Continuous variables were compared using independent samples Student’s t-test. No equal variances were assumed. Correlations were calculated using Pearson correlation coefficient. The tests used were two-sided, and p-values < 0.05 were considered significant.

## Results

Volumetric data and tagging results are presented in Table 1. On average, systolic and diastolic volumes and ejection fractions of both ventricles were within normal range. Of the patients, 60% had increased septal LV wall thickness and 16% had increased LV EDV (> 100 ml/m^2^). Apical rotation (8.6° ± 3.7°) was higher than basal rotation (− 3.5° ± 2.4°), as anticipated.

**Table 1:** Volumetric and tagging results of the study population (n = 50)

Parameter	Value
LV EDV (ml/m^2^)	85.4 ± 15.1
LV ESV (ml/m^2)^	34.8 ± 12.0
LV EF (%)	60.9 ± 6.9
LV mass (mg/m^2^)	60.9 ± 6.9
RV EDV (ml/m^2^)	81.3 ± 16.1
RV ESV (ml/m^2^)	30.4 ± 9.5
RV EF (%)	64.4 + 6.6
HR (bpm)	68 ± 11
Hematocrit (%)	39.6 ± 3.4
Basal rotation (°)	− 3.5 ± 2.4
Apical rotation (°)	8.6 ± 3.7
Torsion (°/mm)	0.43 ± 0.13

*LV* left-ventricle, *RV* right-ventricle, *EDV* end-diastolic volume, *ESV* end-systolic volume, *EF* ejection fraction, *HR* heart rate

### Strain findings

Strain and strain rate values for different LV segments and mean values are presented in Table 2. At the basal level, inferoseptal and inferior peak circumferential strain (CS) were significantly lower than other segments (half or more; p < 0.001) at this level. Similarly, peak radial strain (RS) of anteroseptal and inferoseptal segments were significantly lower than other basal segments (p < 0.005). The peak systolic and diastolic radial strain rates (RSR) of these segments were also significantly reduced relative to other basal segments (p < 0.001). The peak systolic and diastolic circumferential strain rates (CSR) were lower in some of the basal segments, but not as significantly as in the radial direction.

**Table 2:** Results for segmental and mean strain and strain rate analysis of both ventricles of the study population (n = 50)

Short-axis	Peak CS (%)	Peak systolic CSR (%/s)	Peak diastolic CSR (%/s)	Peak RS (%)	Peak systolic RSR (%/s)	Peak diastolic RSR (%/s)
LV mean	-19.9 ± 3.4	− 94.4 ± 17.0	86.5 ± 22.4	54.9 ± 10.0	233.0 ± 43.5	− 273.6 ± 59.3
LV base	− 18.2 ± 3.1	− 79.9 ± 14.0	74.0 ± 16.2	51.3 ± 12.7	205.8 ± 55.8	− 235.2 ± 70.4
LV mid	− 18.0 ± 3.4	− 83.0 ± 20.5	79.2 ± 21.2	58.1 ± 11.7	254.2 ± 56.2	− 301.1 ± 85.3
LV apex	− 23.5 ± 5.7	− 120.2 ± 30.6	106.3 ± 40.7	55.4 ± 15.3	239.1 62.8	− 284.5 ± 82.4
RV mean	− 13.7 ± 3.9	− 69.5 ± 19.9	63.7 ± 19.7	NA	NA	NA
RV base	− 11.6 ± 3.7	− 60.9 ± 20.8	46.9 ± 14.3	NA	NA	NA
RV mid	− 14.5 ± 4.7	− 69.4 ± 23.3	65.0 ± 28.9	NA	NA	NA
RV apex	− 15.1 ± 6.6	− 78.0 ± 30.8	79.2 ± 34.8	NA	NA	NA

Long-axis	Peak LS (%)	Peak systolic LSR (%/s)	Peak diastolic LSR (%/s)			
LV mean	− 17.4 ± 2.6	− 79.1 ± 14.5	60.5 ± 14.1			
RV mean	− 22.4 ± 4.2	− 95.8 ± 19.4	67.4 ± 18.7			

LV segments	Peak CS (%)	Peak systolic CSR (%/s)	Peak diastolic CSR (%/s)	Peak RS (%)	Peak systolic RSR (%/s)	Peak diastolic RSR (%/s)
Basal anterior	− 20.8 ± 5.6	− 111.9 ± 32.2	97.5 ± 33.6	55.9 ± 18.9	246.4 ± 79.1	− 278.8 ± 109.4
Basal anteroseptal	− 18.5 ± 6.2	− 90.2 ± 26.6	69.8 ± 27.2	33.5 ± 14.9*	169.2 ± 68.4*	− 174.9 ± 79.4*
Basal inferoseptal	− 14.2 ± 4.9*	− 72.6 ± 20.5	80.6 ± 22.8	26.7 ± 8.9*	134.3 ± 46.0*	− 160.3 ± 50.7*
Basal inferior	− 16.2 ± 5.8*	− 76.4 ± 25.0	78.3 ± 24.8	61.8 ± 19.4	276.2 ± 95.5	− 350.4 ± 127.0
Basal inferolateral	− 21.3 ± 6.7	− 97.3 ± 30.9	128.2 ± 40.4	71.3 ± 16.4	315.4 ± 95.6	− 360.8 ± 124.3
Basal anterolateral	− 21.9 ± 6.6	− 98.6 ± 30.1	112.2 ± 40.0	70.0 ± 19.1	307.7 ± 93.0	− 361.4 ± 128.8
Mid anterior	− 20.6 ± 5.7	− 109.9 ± 23.2	96.6 ± 30.1	64.5 ± 14.4	291.4 ± 63.7	− 382.5 ± 111.2
Mid anteroseptal	− 20.3 ± 5.9	− 114.5 ± 25.7	101.7 ± 30.2	52.0 ± 15.4	271.6 ± 73.6	− 277.4 ± 77.7
Mid inferoseptal	− 21.2 ± 6.5	− 107.5 ± 31.2	114.2 ± 34.3	55.4 ± 15.7	276.2 ± 80.2	− 271.4 ± 95.2
Mid inferior	− 15.3 ± 4.8*	− 71.7 ± 22.7*	86.6 ± 39.4	59.0 ± 13.6	264.2 ± 70.4	− 308.8 ± 94.0
Mid inferolateral	− 19.9 ± 5.3	− 92.3 ± 27.9	118.5 ± 45.6	62.3 ± 13.5	274.4 ± 76.6	− 370.7 ± 138.6
Mid anterolateral	− 14.5 ± 5.1*	− 74.5 ± 27.2*	67.6 ± 33.4*	62.2 ± 14.9	269.7 ± 74.4	− 398.5 ± 151.1
Apical anterior	− 22.8 ± 6.0	− 122.4 ± 33.1	110.7 ± 39.8	59.1 ± 19.9	263.4 ± 86.1	− 330.5 ± 122.0
Apical septal	− 26.9 ± 7.4	− 143.8 ± 37.6	129.5 ± 51.2	44.8 ± 15.7	207.1 ± 69.2	− 249.2 ± 80.2
Apical inferior	− 25.8 ± 7.6	− 127.3 ± 40.0	131.1 ± 49.9	55.5 ± 12.9	259.3 ± 63.0	− 290.6 ± 71.5
Apical lateral	− 19.8 ± 6.3	− 110.7 ± 40.3	98.3 ± 43,2	64.5 ± 19.0	294.7 ± 81.7	− 371.2 ± 126.6

*CS* circumferential strain, *CSR* circumferential strain rate, *RS* radial strain, *RSR* radial strain rate, *LV* left-ventricle, *RV* right-ventricle, *NA* not applicable, *LS* longitudinal strain, *LSR* longitudinal strain rate

*Significantly different (p < 0.05) from other (half or more) segments of the same plane

In the mid-ventricular plane, peak CS and systolic and diastolic CSR were significantly decreased in the inferior and anterolateral segments (p < 0.025). RS or RSR was not significantly different in any segment relative to the others. In the apical imaging plane, there were no significant differences in the segments in any of the strain parameters.

Mean CS values of different planes were similar at basal and mid-ventricular levels in both ventricles, the mean CS was the highest in the apical plane in both ventricles. Mean CSR, RS, and RSR were similar in all planes in both ventricles. The peak longitudinal strain (LS) and systolic and diastolic longitudinal strain rate (LSR) were higher in RV than in LV (p < 0.007).

### T1, ECV, and LGE findings

Native T1, ECV, and LGE values are presented in Table 3. The mean T1 values ranged from 1003 to 1080 ms in different segments of the LV. T1 values were the highest in the basal plane, on average 25 ms higher than in the other planes. Mid-ventricular and apical values were similar, around 1030 ms. Myocardial mean native T1 was 1031 ± 37 ms.

**Table 3:** T1, ECV and LGE results of the study population (n = 50)

Segment	Pre-contrast T1 (ms)	ECV (%)	LGE (N)
Base (mean)	1055 ± 40	30.0 ± 4.4	38
Anterior	1034 ± 43	27.5 ± 4.1	11
Anteroseptal	1078 ± 83	32.0 ± 6.5	25
Inferoseptal	1044 ± 45	30.7 ± 6.1	17
Inferior	1062 ± 37	30.6 ± 5.2	25
Inferolateral	1080 ± 94	31.3 ± 4.6	6
Anterolateral	1031 ± 41	27.7 ± 3.8	4
Mid (mean)	1027 ± 41	27.2 ± 3.2	16
Anterior	1013 ± 50	26.7 ± 3.3	0
Anteroseptal	1017 ± 47	27.4 ± 3.5	3
Inferoseptal	1026 ± 40	26.5 ± 3.3	0
Inferior	1038 ± 50	27.7 ± 4.1	14
Inferolateral	1042 ± 46	27.8 ± 3.7	2
Anterolateral	1026 ± 48	27.2 ± 3.4	1
Apex (mean)	1012 ± 42	28.3 ± 3.3	1
Anterior	1003 ± 49	27.8 ± 3.2	0
Septal	1006 ± 43	27.9 ± 2.8	0
Inferior	1015 ± 50	27.2 ± 3.4	1
Lateral	1022 ± 47	28.3 ± 3.3	0

*ECV* extra-cellular volume, *LGE* late gadolinium enhancement

ECV was below 30% in all mid-ventricular and apical segments. At the basal level, ECV was over 30% in anteroseptal, inferoseptal, inferior, and inferolateral segments. ECV was the highest in the basal anteroseptal segment (32.0% ± 6.5%); in this segment, ECV of over 35% was detected in 19 patients (38%), and the highest single ECV in this segment was 53%. ECV values of different segments correlated significantly with corresponding T1 values (p < 0.005), as expected.

LGE in the basal plane was detected in 38 patients (76% of the study population). LGE was mostly located in the basal septum or RV insertion point, and it was either patchy or subepicardial. Average LGE percentage (in relation to LV mass) was 6 ± 3%. Basal anteroseptal and basal inferior were the segments with the highest amount of LGE detected; 25 patients (50%) had LGE in these segments. At the mid-ventricular level, 14 patients (28%) had LGE in the inferior segment. In other mid-ventricular segments, only single patients had LGE. Only one patient had an apical LGE.

### Correlations of strain parameters with T1, ECV, and LGE

**Fig. 3:**
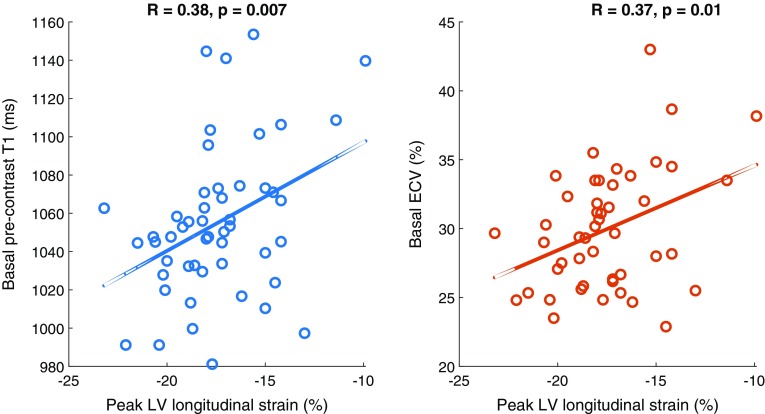
Correlation between peak left-ventricular longitudinal strain and **a** basal pre-contrast T1; **b** basal ECV (n = 50)

Peak LV LS correlated with mean T1 and ECV values of the LV (p < 0.044). The correlations were especially significant in the basal plane (p < 0.01) (Fig. 3). Additionally, LV LS correlated with apical rotation (R = − 0.33, p = 0.018) and LV torsion (R = − 0.39, p = 0.005), meaning weaker peak LS resulted in decreased apical rotation and lower LV torsion. Mid-ventricular LGE also correlated with peak LS (R = 0.34, p = 0.016); LGE decreased peak LS.

In circumferential and radial directions, the only significant correlations were found in the basal plane, between LGE and peak diastolic CSR (R = − 0.48, p < 0.001) and diastolic RSR (R = 0.36, p = 0.01). Increased LGE decreased peak diastolic CSR and RSR. Even though peak CS and RS were significantly decreased in some segments of the LV, no clear correlation in segmental values with T1, ECV, or LGE was found.

## Discussion

This is the first study assessing myocardial motion, T1 relaxation, and ECV of AGel amyloidosis with CMR. We were specifically interested in amyloid accumulation in the myocardium since myocardial involvement is often a prognostic factor in amyloidosis [12]. Our results indicate that hereditary AGel amyloidosis has local myocardial effects, primarily in the basal plane of the left ventricle.

In cardiac amyloidosis, LV volumes and EF are commonly preserved, but LV wall thickness is increased [13]. In our patient population, LV mass was mostly normal, although hypertrophic LV wall was observed in 60% of the patients. Hypertrophy concentrated in the basal ventricular septum. In acquired light-chain (AL) amyloidosis, hypertrophy is often concentric, and on the other hand, prevalence of asymmetrical hypertrophy is more common in hereditary transthyretin-related amyloidosis [13].

Peak strain values of different LV and RV short-axis planes and RV long-axis direction were within normal range [14, 15]. Normal (absolute) peak LV longitudinal strain with FT is reported to be higher than 20% [16]. The absolute peak value of 17.4% in this study indicates decreased longitudinal contraction in the LV. The decreased contraction was associated with decreased apical rotation and thus LV torsion. In our study, LV longitudinal strain also significantly correlated with T1, ECV, and LGE. Previously, peak LV longitudinal strain has been shown to be impaired in patients with cardiac amyloidosis; LGE amount has had a significant impact on longitudinal strain [17]. Previously reported strain values of different LV segments have been within a few percentage points of each other [15]. In this study, specifically the basal anteroseptal, inferoseptal, and inferior segments had significantly decreased circumferential and radial strain values relative to the other segments; the same segments also had the highest amount of LGE in the study population.

Native myocardial mean T1 values were significantly increased (p < 0.0001) when compared with normal myocardial values acquired from a healthy reference population (N = 46; age = 46 ± 9 years; myocardial mean T1 = 971 ± 18 ms) using the same scanner and ShMOLLI pulse sequence. However, the native T1 values were lower than commonly observed in other types of cardiac amyloidosis [18]. Although the T1 values were increased, the ECV was within normal limits at all levels other than basal level [19]. In previous studies focusing on patients with AL and ATTR amyloidosis, the native T1 values have been prolonged, indicating extracellular expansion [18, 20]. No common high T1 or ECV was detected in this hereditary AGel amyloidosis; only single segmental ECV values of over 40% were observed. However, ECV is found to increase markedly in both AL and ATTR types of cardiac amyloidosis, referring to expansion of the interstitium by amyloid deposits [5]. ECV also correlates with disease severity [21].

In addition to elevated ECV, LGE is a common finding in the main types of cardiac amyloidosis. LGE pattern in cardiac amyloidosis may vary and be nonspecific. In AL amyloidosis, global, subendocardial LGE is most common, whereas in ATTR amyloidosis the pattern of LGE is often more extensive and transmural [22]. In our patient group, LGE findings were local, varied, and focused on the ventricular septum and inferiorly. The fact that LGE alone, without significant changes in T1 native and ECV values, except in the areas of LGE, was increased in this study population indicates that the myocardium is fibrotic rather than amyloid.

### Limitations

The study was limited to a relatively minor population for quantitative CMR analysis. Patients with cardiac pacing devices were excluded from the study. As AGel amyloidosis may require pacemaker systems to be implanted in patients during later phases of the disease, the results might have been different at more advanced stages of the disease. Pacemaker devices, however, may impair the image quality in CMR, affecting the reliability of analysis. Furthermore, the patient population selected in this study was over 50 years old and does not represent the entire population of AGel amyloidosis. The selection was made on prior knowledge of disease progression. Intra-observer and inter-observer variability of the results were not assessed. CMR FT has been shown to have inter-software variability in both feasibility and absolute strain values [23]. However, non-rigid elastic registration-based FT used in this study has been demonstrated to be well reproducible, and not be influenced by the level of training of the observer, whether it be 6 months or 20 years [24]. The chosen analysis software of this study employs the same algorithm for both FT strain and tagging image analysis, which increases the technical reliability of the results.

## Conclusions

Contrary to the main types of cardiac amyloidosis, myocardial effects in patients with AGel amyloidosis are mainly local, focused on the basal plane of the left ventricle. Although majority of patients had LGE in this patient population, high increase in T1 or ECV was not detected.
